# Musculoskeletal ultrasonography versus conventional radiography: Correlation with DAS28 and MDHAQ scores in early rheumatoid arthritis

**DOI:** 10.1177/03000605241306397

**Published:** 2024-12-28

**Authors:** Reem Hamdy A Mohammed, Hatem Alazizi, Asmaa Negm Eldin Taha, Seham Metawee

**Affiliations:** 1 Department of Rheumatology and Rehabilitation, RinggoldID:63527 Cairo University, Cairo, Egypt; 2 Department of Radiodiagnosis, School of Medicine, Cairo University, Giza, Egypt

**Keywords:** Early rheumatoid arthritis, functional disability, multidimensional health assessment questionnaire, musculoskeletal ultrasonography, plain radiography

## Abstract

**Objectives:**

To compare the value of musculoskeletal ultrasound (MSUS) with conventional radiography in the detection of patients with early rheumatoid arthritis (RA) and to correlate the sonographic findings with disease activity, and functional disability scores.

**Methods:**

Patients >18 years of age with RA ≤2 years who satisfied the 2010 EULAR/ACR classification criteria for rheumatoid arthritis and disease activity score 28 (DAS28) >2.6, were enrolled. Plain radiographs and MSUS examinations were performed on 18 joints bilaterally. DAS28 and multi-dimensional health assessment questionnaire (MDHAQ/RAPID) scores were assessed.

**Results:**

Forty patients (35 women, 5 men), mean age 41 ± 12 years, and mean disease duration 11 ± 5 months, were included. In total, 720 joints were examined. The number of hand joints affected by erosions via MSUS was 3.28-fold the number detected by X-ray. Sonographic evidence of synovitis and active erosion significantly correlated with MDHAQ, DAS28 and inflammatory biomarkers.

**Conclusion:**

Joint sonography was superior to conventional radiography in early detection of structural joint damage and active disease in patients with early RA which correlated with disease activity and functional ability scores.

## Introduction

Rheumatoid arthritis (RA) is a chronic multisystem autoimmune disease that primarily affects the synovial lining of the diarthrodial joints with ongoing inflammation and progressive disability.^1^ Reported prevalence rates range from 0.5% to 1% with regional variations, and women are more frequently affected than men, with a predominance in the elderly population.^2^ A significant shift in the management paradigm of RA has occurred over the past two decades, with a move towards early diagnosis followed by prompt treatment and close follow-up aiming at long-term remission.^3^ Early diagnosis in RA has proved to be key for a successful outcome in terms of reduced joint destruction, suppression of radiographic progression, and prevention of functional disability. These positive effects have been associated with a simultaneous improvement in the cost effectiveness of therapy.^4^

The increasing use of musculoskeletal ultrasound (MSUS) in the field of diagnostic imaging in rheumatology has significantly supported best clinical practice. This imaging modality can be used as a bedside diagnostic imaging tool to detect early inflammation, assess disease activity, and monitor ongoing damage; it can assist in timely decision making which will improve outcomes and prevent progressive disability. In addition, MSUS has demonstrated a promising predictive value in the development of clinical arthritis in patients with subclinical disease or palindromic attacks. MSUS can also be used to evaluate the effects of therapeutic interventions on disease processes and prognostics.^5^

The primary objective of the present study was to compare the value of MSUS as a diagnostic imaging tool with conventional radiography in the detection of patients with early RA according to the 2010 EULAR/ACR classification criteria.^6^ A secondary objective was to correlate the sonographic findings with disease activity, and functional disability scores.

## Methods

### Study design and patients

Patients >18 years of age with RA, who satisfied the 2010 EULAR/ACR classification criteria,^6^ were consecutively recruited from the Rheumatology and Rehabilitation Outpatient clinic, School of Medicine, Cairo University Hospital, over a period of 12 months (2016). Eligibility criteria have included patients with early onset RA (i.e., disease duration ≤2 years) and had a disease activity score 28 (DAS28) >2.6.^7,8^ Patients with juvenile idiopathic arthritis, other autoimmune diseases or overlap syndromes, endocrinopathies, haemoglobinopathies or coagulopathies were excluded from the study. Medication dosages remained unmodified during the assessment period for the RA patients.

The reporting of this study conforms to STROBE guidelines.^9^ The study obtained formal approval by the Ethics Committee of School of Medicine Cairo University. Written informed consent was obtained from patients and controls and patient data were anonymized prior to analysis.

### Data collection

Detailed history, clinical examination, laboratory workup tests and radiographic findings were performed on all patients ≤3 weeks before MSUS to avoid temporal dissociation and faulty interpretation of data.

Patients were assessed in terms of DAS28 which is based on a count of 28 swollen and tender joints, erythrocyte sedimentation rate (ESR), and global health (GH) assessment. It ranges from 0 to 9.4, and, is calculated as follows:^8^ DAS 28 = (0.56 × √ [tender 28] + 0.28 √ (swollen 28) + 0.70 ln (ESR) + 0.014 (GH). Level of disease activity was interpreted as low (≤3.2) moderate (>3.2 to ≤5.1) or high (>5.1).^8^ Patients with scores score <2.6 were regarded as having inactive disease or were in remission.

Functional status was assessed using the multidimensional health assessment questionnaire (MDHAQ) which includes RAPID3 (routine assessment of patient index data).^10^ RAPID3 is composed of three self-report scores for physical function (FN), pain (PN] and patient global estimate (PTGL), each scored 0-10, for a total of 0-30. Higher scores indicate poorer status. Four severity categories have been proposed: high (>12), moderate (>6–12), low (>3–6) and near remission (≤3).^11^ Two additional RAPID based scores were also assessed: RAPID 4 adds a self-report joint count, and RAPID 5, a physician global estimate.^12^

In addition, patients had plain radiographs (X-rays) taken of hands and wrists in a posteroanterior (PA) view. Erosions and joint space narrowing were scored using a modified version of Sharp erosion score (van der Heijde modification).^13^ The scoring was as follows: grade 0, normal grade 1, discrete erosions; grade 2 to 3, larger erosions according to surface area involved; grade 4, erosions extending over middle of the bone; grade 5, complete collapse.^13^

Ultrasonography was performed in accordance with EULAR standard scanning techniques^14^ by an experienced sonographer who was blinded to the clinical and radiographic results. Scans were performed on all subjects using General Electric Logiq P5 ultrasound machine with a near focused linear array transducer and frequency of 7 MHz to 13 MHz. Ultrasound examinations of soft tissue and bone changes (grey scale (GS) imaging and power doppler [PD]) were performed on 18 joints bilaterally (2^nd^ to 5^th^ metacarpal-phalangeal [MCP] joints, 2^nd^ to 5^th^ proximal interphalangeal joints [PIP] and wrist) according to OMERACT definitions of ultrasonic pathology.^15^ The EULAR- OMERACT semi-quantitative scoring scale for assessment of synovitis was expressed per joint.^15,16^ The highest grade for synovial hypertrophy (SH) or PD detected in any of the examined joints was used as representative for that patient.

According to GSUS imaging, joints were graded 0–4 as follows: 0, normal joint (no synovial hypertrophy, no joint effusion); 1, minimal synovitis (minimal synovial hypertrophy, with or without minimal joint effusion); 2, moderate synovitis (moderate synovial hypertrophy with or without minimal or moderate joint effusion); 3, severe synovitis (severe synovial hypertrophy, with or without severe joint effusion). Using PDUS, joints were graded 0–3 as follows: 0, no vessel in the synovium; 1, up to 3 single spots signals or 1 confluent spot + up to 2 single spots; 2, vessel signals in less than half of the area of the synovium (<50%); 3, vessel signals in more than half of the area of the synovium (>50%).

### Statistical analysis

Statistical analysis was performed using SPSS software (version 15.0 for Windows^®^; SPSS Inc., Chicago, IL, USA). A *P*-value <0.5 was considered to indicate statistical significance. Data were described in terms of mean ± standard deviation (SD), median and range, or frequencies and percentages as appropriate. Comparison of numerical variables between study groups was performed using Mann Whitney *U* test for independent samples. Categorical variables were compared using χ^2^ tests and Fisher's exact test was used when the expected frequency of one or more cells was <5.

Spearman rank correlation analysis was used to determine associations between ultrasound results and several clinical variables.

## Results

### Patients

Forty patients (35 women, 5 men), mean age of 41.0 ± 12.2 years and mean disease duration 11 ± 5 months were included in the study. Six (15%) RA patients were not receiving treatment. Of the remaining 34 patients (85%), 19 (48%) were receiving non-steroidal anti-inflammatory drugs (NSAIDs), 32 (80%) were receiving methotrexate (12.5–25 mg/w [19.7 ± 3.2 mg/w]), 6 (15%) were receiving hydroxychloroquine (200–400 mg/d [320 ± 109 mg/d]), and one patient (3%) was receiving leflunomide 20 mg/d and 18 patients (45%) on oral corticosteroids (5–10 mg/d [6.3 ± 2.0 mg/d]).

In total, 720 joints were examined using MSUS. Clinical evaluation showed that arthritis of the small joints of the hands was the first presentation in 35 (88%) patients and tenosynovitis was detected in 5 (13%) patients. Overall, the mean swollen joint count was 1.75 ± 2.18, and mean tenderness score was 3.22 ± 3.23.

Laboratory work-up showed ESR was elevated in 15 (38%) patients, C reactive protein (CRP) was elevated in 26 (65%) patients and anemia was present in 7 (18%) patients. Rheumatoid factor (RF) was positive in 21 (53%) patients and negative in 19 (48%). Twenty-two patients had blood samples tested for anti-citrullinated peptide antibodies (ACPA) of whom 12 (55%) were positive.

### Disease activity and functional assessment

DAS28 scores ranged from 2.6 to 7.6 with a mean of 4.2 ± 1.3. According to these scores, 15 (38%), 18 (45%) and 7 (18%) patients were assessed as having low (≤3.2), moderate (>3.2 to ≤5.1) or severe (>5.1) disease activity.

MDHAQ scores for physical function ranged from 0–10 (mean 3.5 ± 2.9), pain ranged from 2–10 (mean 5.7 ± 2.2), global status ranged from 1–10 (mean 4.5 ± 2.8), self-report joint count status ranged from 0.2–10 (mean 1.9 ± 1.9) and physician global estimate ranged from 2–8 (mean 2.6 ± 2.9).

RAPID3 scores (0–30) ranged from 4–29 (meaning 13.4 ± 6.2). With respect to functional impairment, 5 (13%) patients were categorized as low severity (>3 and ≤6), 16 (40%) as moderate severity (>6 and ≤12) and 19 (48%) as severe (>12).

RAPID4 (0–40) scores ranged 4.2–36 (mean of 15.3 ± 7.7). With respect to functional impairment, 6 (15%) patients were categorized as low severity (>4 and ≤8), 22 (55%) as moderate severity (>8 and ≤16) and 12 (30%) patients as severe (>16).

**Figure 1.**
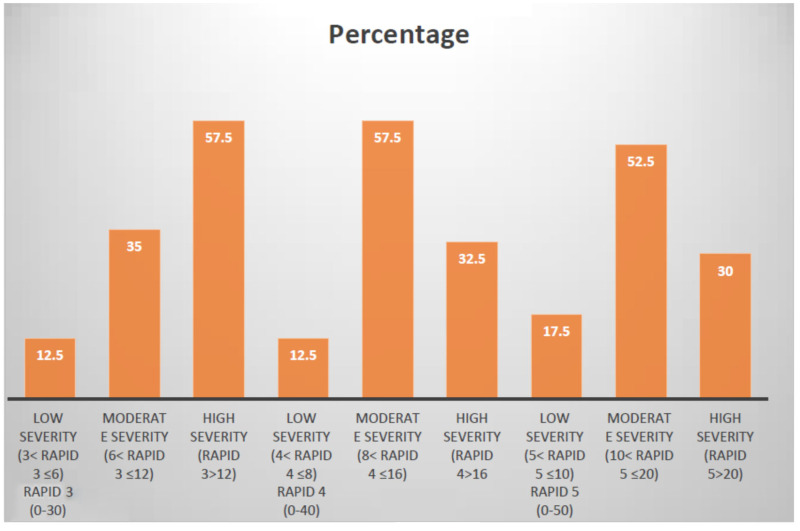
Results of RAPID composite scores.

RAPID5 (0–50) scores ranged from 6–44 (mean 17.9 ± 9.7). With respect to functional impairment, 7 (18%) patients were categorized as low severity (>5 and ≤10), 21 (53%) as moderate severity (>10 and ≤20) and 12 (30%) patients as severe (>20). Figure 1 shows the results of RAPID composite scores.

### Radiographic results

Radiographic assessments of patients’ hands showed the following: juxta articular osteopenia in 35 (85%) patients; soft tissue swelling in 16 (40%) patients; cystic changes in 4 (10%) patients; bone erosions in 3 (8%) patients. Erosions were observed in 3 (8%) patients and in 7/720 (0.97%) joints including 2 erosions in the wrists, and 5 in the MCP joints. No erosions were detected in the PIP joints. Using Van der Heijde modification of the sharp erosion score,^13^ scores ranged from 0–4 (mean, 1.29 ± 0.48).

### Ultrasound results

Ultrasound examinations showed erosive disease in 7 (18%) patients and 23/720 (3%) of assessed hand joints. MSUS showed that erosions were detected in 54 (8%) of wrist joints, 30 (4%) of MCP joints and 9 (1%) of PIP joints.

Synovial hypertrophy (SH) was detected in 100% patients and in 178/720 (25%) joints. Numbers of patients with mild, moderate and severe SH were, 6 (15%), 25 (63%) and 9 (23%) patients, respectively. Positive PDUS signals for active synovitis were observed in 25 (63%) patients and 86/720 (12%) joints. Using the PDUS semi-grading scale, 8 (20%), 14 (35%) and 3 (8%) patients were graded as 1, 2 and 3, respectively.

According to GSUS assessment, tenosynovitis was observed in 11 (28%) patients, of whom, 6 (15%) also had a positive PDUS signal. Of these 11 patients, extensor tenosynovitis was detected in 7 (18%) patients, of whom, 2 (5%) had a positive PDUS signal which indicated active inflammation. In addition, flexor tenosynovitis was detected in the remining 4 (10%) patients, and all had a positive PDUS signal which indicated active inflammation.

**Figure 2.**
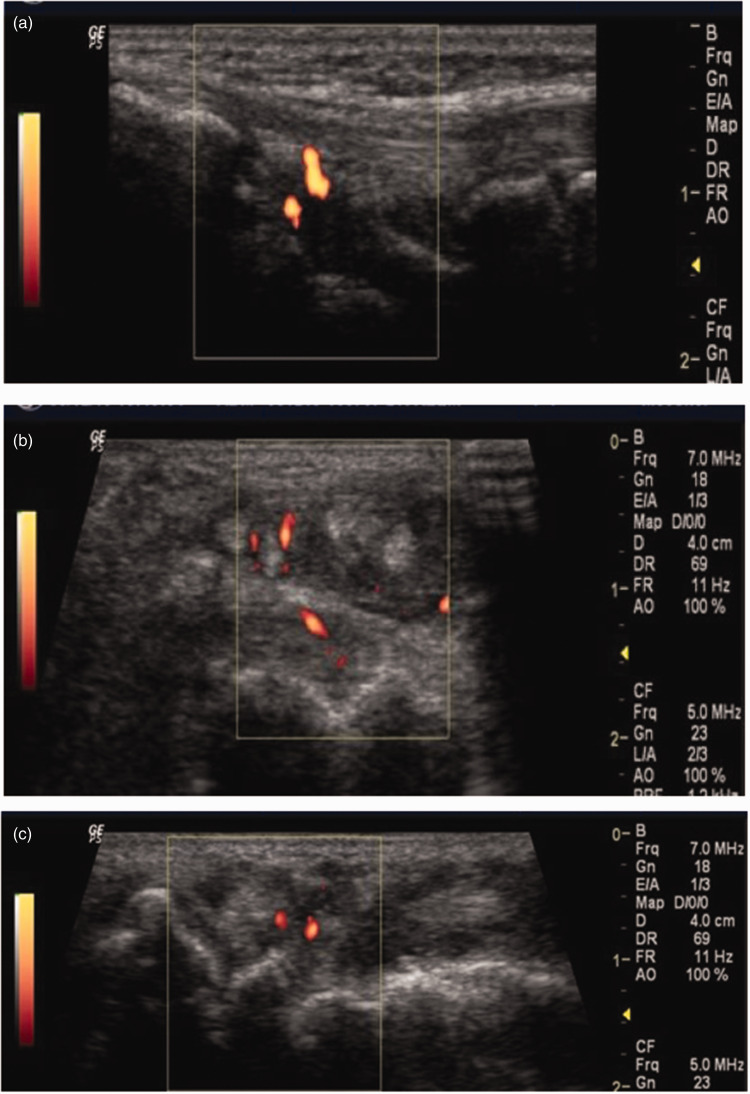
(a) Dorsal longitudinal scan of the right wrist joint using 7–13 MHz linear transducer showing synovitis with positive doppler signal. (b) Transverse dorsal scan showing peri-tendonitis and synovitis grade II doppler signal with irregular cortical surface of the carpal bones (erosive changes) and (c) Dorsal transverse scan showing synovitis, effusion and erosive changes in the cortical surface of the carpal bones.

Sonographic images of a wrist joint showing synovitis, peri-tendonitis and synovitis with erosive changes and synovitis and erosive changes are shown in Figure 2(a), (b) and (c), respectively.

**Figure 3.**
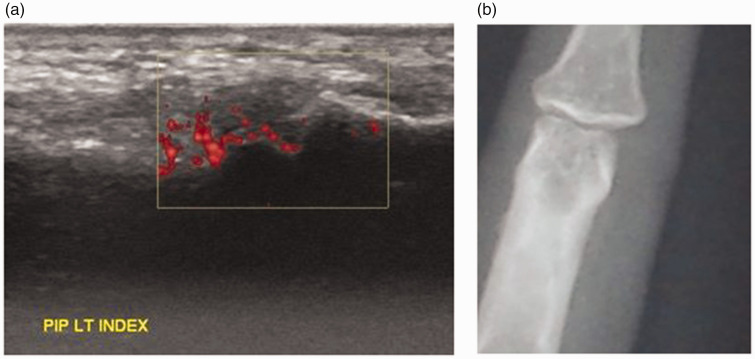
(a) Ultrasound of the 2nd PIP joint palmar longitudinal view with hypoechoic area denoting severe synovial hypertrophy grade 3 with moderate vascularization (grade 2 power Doppler signal) denoting moderate inflammation and (b) Plain radiograph of the 2nd PIP joint taken in a posteroanterior (PA) view showing juxta articular osteopenia.

### Comparison of radiographic and ultrasound results

Of 720 hand joints examined using MSUS, erosions were detected in 23 (3%) hand joints and in 7 (18%) patients compared to conventional radiography, where erosions were detected in 7 (1%) hand joints and in 3 (8%) patients. Therefore, the number of hand joints with erosions by MSUS was 3.28-fold the number detected by conventional radiography. Similarly, the number of patients with erosions in hand joints detected by MSUS was 2.33-fold more than the number detected by X-ray. These differences were statistically significant (*P* = 0.006). A comparison of radiographic and ultrasound images of the 2nd PIP joint shown in Figure 2.

### Correlation of ultrasound results with several clinical variables

Positive GSUS and PDUS images for synovitis and active erosions significantly correlated with RAPID 3, RAPID 4 and RAPID5 scores (Table 1). They also correlated with DAS28 scores. Synovitis by GSUS and PDUS significantly correlated with the duration of morning stiffness. In addition, synovitis identified by ultrasound correlated with the inflammatory biomarkers ESR and CRP but the presence of erosions did not correlate with the biomarkers. There was no significant correlation between sonographic findings and disease duration, positive RF, age, or sex (Table 1).

**Table 1.** Correlation between ultrasound findings and several clinical variables.

Variables	Grey scale ultrasound results		Power Doppler ultrasound results (Synovitis)		Erosions	
	r	Statistical significance	r	Statistical significance	r	Statistical significance
RAPID3	0.60	*P* = 0.0003	0.47	*P* = 0.002	0.39	*P* = 0.01
RAPID4	0.60	*P* = 0.0003	0.46	*P* = 0.002	0.41	*P* = 0.009
RAPID5	0.62	*P* = 0.0001	0.57	*P* = 0.0006	0.41	*P* = 0.008
DAS28	0.61	*P* = 0.0001	0.73	*P* = 0.0001	0.36	*P* = 0.02
ESR	0.48	*P* = 0.02	0.71	*P* = 0.0006	0.21	ns
CRP	0.50	*P* = 0.001	0.80	*P* = 0.0001	0.18	ns
Morning stiffness	0.56	*P* = 0.0002	0.80	*P* = 0.0001	0.30	ns
RF	0.11	ns	−0.11		0.043	ns
Disease duration	0.11	ns	−0.02	ns	0.04	ns
Age	0.14	ns	0.01	ns	−0.07	ns
Sex	0.188	ns	0.146	ns	0.174	ns

RAPID, routine assessment of patient index data; DAS28, disease activity score 28; ESR, erythrocyte sedimentation rate; CRP, C-reactive protein; RF, rheumatic factor; ns, not statistically significant.

A significantly high incidence of SH was detected in patients with erosive disease identified by MSUS (*P* = 0.017). According to DAS28, a positive PD signal was observed in 100% patients with a high score, 83% patients with moderate score, and 20% patients with low score (*P* = 0.001). Interestingly, there was a significant difference in the PD signal (synovitis) between RA patients on basic disease-modifying antirheumatic drugs (DMARDs; 34 [85%]) and DMARD naïve patients (6 [15%]; *P* = 0.002). Similarly for erosions, MSUS examinations showed a significantly lower incidence of erosions in patients on methotrexate (2/32, 6%) compared with those not on methotrexate (5/12, 42%; *P* = 0.017).

## Discussion

Early diagnosis of RA with effective control of inflammation demands a constellation of sensitive and specific methods for monitoring disease activity and measuring damage.^13^ While conventional radiography is the most widely used imaging modality for RA because of its wide availability, it is associated with some limitations including; low sensitivity for assessment of early disease, hazards of ionizing radiation, two-dimensional representation of a three-dimensional pathology and insufficiency of assessment of soft tissue abnormalities including synovitis.^17–19^ MSUS has now become an approved bedside imaging modality that can aid early diagnosis and treatment decision in patients with RA and has been deemed as an appropriate adjunct in the evaluation of RA by the American College of Rheumatology (ACR) and the European League Against Rheumatism (EULAR).^6^

The primary objective of the present study was to compare the value of MSUS as a diagnostic imaging tool with conventional radiography in the detection of patients with early RA and to correlate the sonographic findings with the disease activity, and functional disability scores. We found that MSUS detected bone erosions in a significantly larger number of joints compared with conventional radiography by 3.28-fold and in a greater number of patients by 2.33-fold. Although slightly lower, our findings were consistent with those from a study by Wakefield et al., that found joint sonography identified 6.5-fold more erosions than radiography and 7.5-fold more patients with early onset RA.^20^ The difference in the results between the two studies may have been attributed to different population characteristics (RA patients were not on basic DMARDS), in addition to the previous use of 3D-ultrasonography and ‘hockey stick’ transducer in the earlier study. Similarly, another study found significantly more erosions in MCP and metatarsal-phalangeal (MTP) joints (17/80, 21%) by ultrasound compared with radiography (6/80, 8%).^21^ Although the previous results were in accordance with our findings, their detection rate by both modalities was higher than ours which was possibly due to the long-standing disease duration of the patients in their study (i.e., 50% of their patient had RA for >5 years). Other research conducted in different countries has demonstrated the advantages of ultrasonography over conventional radiography in identifying early arthritis.^22–24^ To our knowledge this current study is the first Egyptian study to explore the difference and unveil such queries.

Validated outcome measures in RA have included several questionnaire-based metrics (e.g. MDHAQ, RAPID3, and DAS28). We used the MDHAQ/RAPID scores as a measure of functional disability and correlated results with ultrasonographic findings. Our results showed a significant correlation between functional incapacitation scores and ultrasonographic synovitis and erosion using GS and PD a finding that is in accord with another study that investigated the diagnostic value of MSUS in 31 newly diagnosed patients with RA and found that PD synovitis total score correlated well with morning stiffness.^24^

Our current study also found a significant correlation between DAS28 and ultrasonographic findings. In this respect, our results are consistent with findings from another study by Mitran et al., using inpatients with early RA that reported a statistically significant positive correlation between GSUS and PDUS synovial inflammation and DAS28-ESR.^25^ Similarly, in another study, the relationship between ultrasound findings (GS and PD) and DAS-ESR was found using dorsal and palmar approach to the finger joints.^26^ In addition, Vlad et al., have shown a significant correlation between functional incapacitation using the health assessment questionnaire (HAQ) and ultrasound score and a significant correlation between the ultrasound echogenicity score and DAS-28.^27^

Interestingly, clinical evidence suggests that early diagnosis and control of RA by use of DMARDs reduces radiographic damage.^28^ We found that the PD signal was significantly lower in patients receiving methotrexate compared with other patients. Similarly for erosions, MSUS examinations showed a significantly lower incidence of erosions in patients on methotrexate compared with those on other drugs. Witt et al., study reported that PDUS findings lowered significantly between baseline and 6-month follow up on both palmar and dorsal approaches in patients with early RA on methotrexate therapy.^26^ Similarly, other studies have demonstrated the efficacy of methotrexate in RA on radiographic signs of active disease.^29–31^

Our study had some limitations. Firstly, the relatively small sample size. Secondly, the ultrasound machine we used had a relatively low resolution with a GS (B-mode) frequency of 7–13 MHz, since it represents a real-world machine that we use in daily practice for patient assessment. Finally, most of our patients were on at least two drugs which may have affected our results. However, this shortcoming is difficult to control in real life clinical practice and this was handled in our study by performing statistical adjustment for potential confounders. Further, large-scale studies are highly recommended to elaborate more data in the field and promote accurate quantitative assessment of RA joint disease via MSUS.

In conclusion, results of our study supported the superiority of ultrasonography over conventional radiography in the evaluation of patients with early RA. MSUS detected more joints with erosions than conventional radiography. Moreover, Sonographic findings of active synovitis and active erosions significantly correlated with MDHAQ/RAPID scores, DAS28 and inflammatory markers. We recommend that ultrasound examination should accompany clinical and laboratory assessment in routine practice seeking precision in disease assessment and management.
